# Enteroviruses: A Gut-Wrenching Game of Entry, Detection, and Evasion

**DOI:** 10.3390/v11050460

**Published:** 2019-05-21

**Authors:** Alexandra I. Wells, Carolyn B. Coyne

**Affiliations:** 1 Department of Pediatrics, University of Pittsburgh School of Medicine, Pittsburgh, PA 15213, USA; ALW214@pitt.edu; 2 Center for Microbial Pathogenesis, UPMC Children’s Hospital of Pittsburgh, Pittsburgh, PA 15224, USA; 3 Richard K. Mellon Institute for Pediatric Research, UPMC Children’s Hospital of Pittsburgh, Pittsburgh, PA 15224, USA

**Keywords:** enteroviruses, gastrointestinal tract, pattern recognition receptors, interferon

## Abstract

Enteroviruses are a major source of human disease, particularly in neonates and young children where infections can range from acute, self-limited febrile illness to meningitis, endocarditis, hepatitis, and acute flaccid myelitis. The enterovirus genus includes poliovirus, coxsackieviruses, echoviruses, enterovirus 71, and enterovirus D68. Enteroviruses primarily infect by the fecal–oral route and target the gastrointestinal epithelium early during their life cycles. In addition, spread via the respiratory tract is possible and some enteroviruses such as enterovirus D68 are preferentially spread via this route. Once internalized, enteroviruses are detected by intracellular proteins that recognize common viral features and trigger antiviral innate immune signaling. However, co-evolution of enteroviruses with humans has allowed them to develop strategies to evade detection or disrupt signaling. In this review, we will discuss how enteroviruses infect the gastrointestinal tract, the mechanisms by which cells detect enterovirus infections, and the strategies enteroviruses use to escape this detection.

## 1. Introduction

### 1.1. Enteroviruses

According to the Centers for Disease Control and Prevention, enteroviruses cause at least 10–15 million symptomatic infections in the United States each year [1]. These viruses belong to the *Picornaviridae* family and are small, non-enveloped viruses that have a single stranded positive sense RNA genome. The enterovirus genus includes poliovirus (PV), coxsackieviruses, echoviruses, enterovirus 71 (EV71), enterovirus D68 (EV-D68), and rhinoviruses. These viruses are spread primarily through the fecal–oral route, but some species can be spread through respiratory secretions (e.g., EV-D68 and rhinovirus). Nonpolio enteroviruses are typically asymptomatic or cause minor clinical symptoms which include hand-foot-and-mouth disease and respiratory illness. In some cases, enteroviruses can cause severe complications which include acute flaccid myelitis, myocarditis and encephalitis, pancreatitis, hepatitis, and even death [2,3,4,5].

The pediatric and neonatal populations can develop severe symptoms and grave clinical outcomes of enterovirus infections [6,7,8]. In fact, enteroviruses are one of the top viral pathogens that cause outbreaks in neonatal intensive care units (NICUs) across the United States each year [9,10], and infections in infants and neonates are associated with high morbidity and mortality. Additionally, enterovirus infections particularly impact young children during outbreaks, as seen in the EV71 outbreak in China from 2008 to 2012. This outbreak was responsible for over 7 million infections with a majority of infections in children under the age of five [11]. In addition to EV71 outbreaks, EV-D68 outbreaks have been intensifying throughout the world with outbreaks in 2014, 2016, and 2018 [12]. EV-D68 outbreaks also typically impact neonates and children and have caused many cases of acute flaccid myelitis (AFM) in the United States, including 120 confirmed cases across 34 states in 2014 [13,14]. Although these severe outcomes are the focus of many studies, most individuals infected with enteroviruses are asymptomatic. Overall, enteroviruses are a significant public health concern, particularly in the pediatric population, due to severe complications from infection in children and neonates.

The immune response to enteroviruses is imperative for successful host clearance. A sufficient immune response to clear enterovirus infection includes the activation of innate immune signaling and a strong B cell response. The antibody response can be extremely important to clear an enterovirus infection. Previous studies have shown that about 50% of adults and older children have neutralizing antibodies against at least two non-polio enteroviruses and over 75% of adults and children have neutralizing antibodies to PV serotypes [15,16]. Neutralizing antibodies to PV arise from vaccination-induced long-lived memory B cells and neutralizing antibodies that are protective against infection [17,18]. This suggests that neutralizing antibodies are important for protection from re-exposure and may explain why children and neonates are most likely to experience severe infection since they likely lack these antibodies [15]. Consistent with this, individuals with X-linked agammaglobulinemia, where the patient has little to no B cells, are highly susceptible to enterovirus infection [19,20]. In addition, mice deficient in B cells have high coxsackievirus B (CVB) titers in their tissues and experience chronic infection and an inability to clear the virus [21]. Thus, the concerted actions of the innate and adaptive immune response allow for the clearance of enteroviruses.

### 1.2. The Gastrointestinal Tract

**Figure 1:**
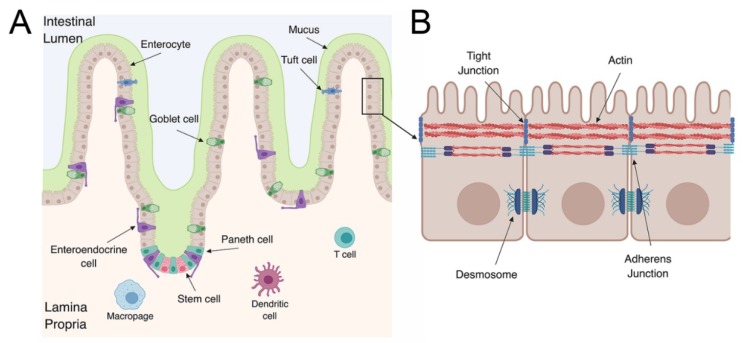
The gastrointestinal tract defenses. (**A**) The gastrointestinal tract is composed of numerous cell types that are important for immune activation and barrier surface defenses. The gastrointestinal epithelium is composed of enterocytes, goblet cells, Paneth cells, enteroendocrine cells, tuft cells, and stem cells. In contrast, the lamina propria is composed of immune cells such as dendric cells, T cells, and macrophages. (**B**) Polarized intestinal epithelial cells have distinct apical and basolateral domains. The apical domain contains microvilli and is closely associated with the actin cytoskeletal network.

The gastrointestinal (GI) tract is a key defensive barrier against pathogenic bacteria and viruses. The GI tract is divided into different subsections: The duodenum, jejunum, and ileum, which make up the small intestine, large intestine, and colon. The GI tract is composed of an epithelial layer that forms a physical cellular barrier as well as a lamina propria that contains immune cells [22]. The lamina propria is essential to elicit an adaptive immune response to pathogens that breach the epithelium. This region contains dendritic cells and macrophages that are able to present viral antigens as well as many other immune cells that are important for initiating a cellular immune response (Figure 1A). In addition to these two compartments, specialized subsections of the epithelium and the lamina propria contain Peyer’s patches. Peyer’s patches contain organized lymphoid structures that sample the intestinal lumen to initiate mucosal immune responses. The formation and role of Peyer’s patches in mucosal immunity have been extensively reviewed elsewhere [23,24,25].

The GI tract, like many barrier surfaces, has important defense mechanisms to prevent microbial invasion. The cells that comprise the epithelium are polarized, meaning they have distinct apical and basolateral surfaces that contain distinct lipid and protein components. The apical surface of enterocytes, which make up a high proportion of the epithelium, contain microvilli that create a dense brush border. The GI epithelium forms a physical barrier due to junctional complexes composed of tight and adherens junctions as well as desmosomes (Figure 1B) [26]. These junctional complexes are important for restricting the free flow of ions and solutes [27]. In addition, differentiated enterocytes have a dense cortical actin network that is critical for preventing pathogens from gaining access to the subcellular domain [28]. Finally, the epithelium utilizes chemical defenses and secretes type I and III interferons (IFNs) to trigger an antiviral state during viral infections [29,30,31]. The concerted actions of the epithelium and the cells that comprise the lamina propria are essential in the defense against enteric pathogens.

The GI epithelium has villus and crypt structures that influence the morphology of the intestine (Figure 1A). The villi project into the intestinal lumen and are mainly composed of enterocytes with other cell types scattered throughout the villi. The base of the crypts contain stem cells that are responsible for the renewal of all the cell types of the GI epithelium [32,33]. The LGR5+ stem cells at the base of the crypt are lined by Paneth cells, which are critical for maintaining stem cell homeostasis (growth factor production) and the secretion of antimicrobial peptides [34,35]. Other cell types are also critical for stem cell differentiation, including crypt-specific fibroblasts [36]. In addition to stem cells and Paneth cells, the epithelium is composed of at least six distinct cell types that help execute the barrier’s essential functions. The cell types can be broken into two main subgroups: those of absorptive and secretory lineages. The absorptive lineage is comprised mainly of enterocytes and microfold (M) cells. M cells are grouped into the absorptive lineage due to their role as conduits between luminal contents and immune cells in the lamina propria and Peyer’s patches [37]. The secretory lineage includes enteroendocrine cells, Paneth cells, and goblet cells [38]. As the name suggests, cells comprising the secretory lineage mainly secrete proteins into the lumen of the GI tract. Goblet cells produce and secrete mucus, which covers the epithelium and has a protective function against pathogens [39]. On the other hand, enteroendocrine cells produce hormones that are thought to provide signals to stem cells. Each cell type is responsible for separate functions to maintain homeostasis of the GI epithelium. Without each cell type, the delicate balancing act of protecting against pathogens, maintaining correct equilibrium with the microbiome, and the absorption of nutrients would be disrupted.

## 2. Enterovirus Infections in the GI Tract

Enteroviruses are primarily transmitted through the fecal–oral route and target the GI epithelium. Enteroviruses are typically ingested through contact with contaminated surfaces, food, and/or water. These viruses are not thought to cause gastrointestinal illness such as severe vomiting or diarrhea, but GI-associated complications may occur [40]. However, most cases of enterovirus infection are asymptomatic [41]. Once they infect the GI epithelium, enteroviruses can disseminate into secondary target tissues and can cause clinical disease in some cases. Enteroviruses have specific secondary tissue tropism that vary between enterovirus species. EV71 has been shown to disseminate into the skin and brain causing hand-foot-and-mouth disease and aseptic meningitis or acute flaccid myelitis, respectively [42]. On the other hand, coxsackievirus B (CVB) can disseminate to the heart and pancreas to cause myocarditis and pancreatitis [5,43,44]. Additionally, echoviruses target the liver as well as the brain causing acute liver failure and aseptic meningitis [4,45]. Despite these differences in secondary target tissues, most enteroviruses, with the exception of rhinoviruses and EV-D68, replicate in the GI epithelium. Enteroviruses target the epithelium for replication and; therefore, this barrier surface is an important defense mechanism for preventing the dissemination of these viruses into secondary target tissues.

**Figure 2:**
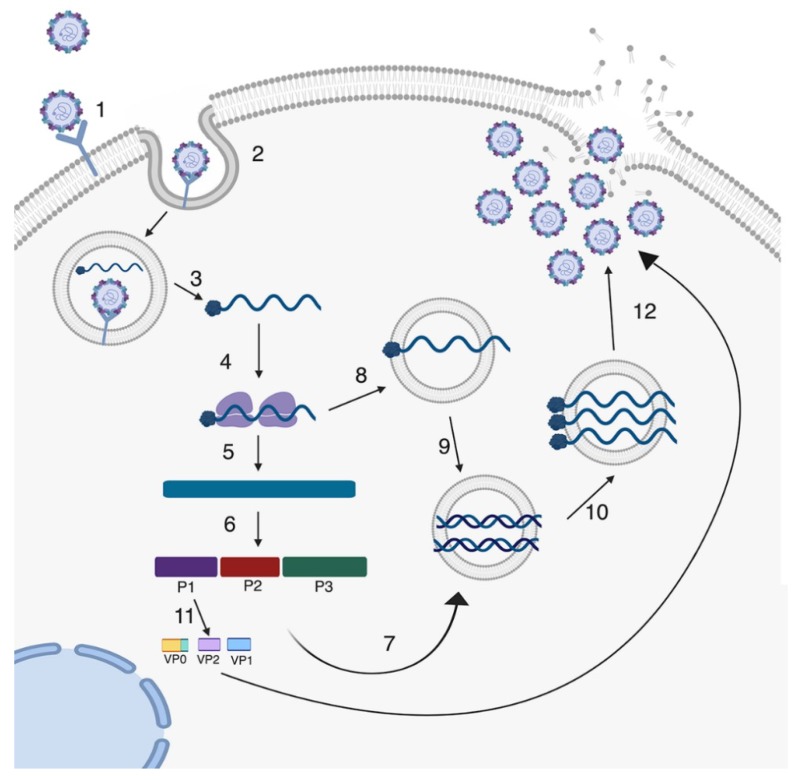
Enterovirus life cycle. Enteroviruses enter the cell through receptor-mediated endocytosis (1). Following endocytosis, uncoating of the virion occurs in the endosome and the positive-stranded RNA along with the covalently-linked VPg protein is released into the cytoplasm (2 and 3). Viral RNA is translated by host ribosomes making a single polyprotein that is catalytically cleaved by enterovirus proteases 2A^pro^ and 3C^pro^ (4, 5, and 6). After production and accumulation of non-structural proteins, including the viral polymerase, viral RNA is then replicated using the virally-encoded RNA-dependent RNA polymerase to generate a double-stranded RNA (8 and 9). The negative sense RNA serves as the template to make more positive sense RNA. This newly produced RNA can be the template to produce more positive sense RNAs or serve as the genome for progeny viruses (10). Capsid proteins assemble and newly synthesized positive-stranded viral RNA is packaged into virion (11). Finally, new progeny virions are released either by non-lytic release, where virions are released in vesicles (not shown), or are released when the cell undergoes lysis (lytic release) (12).

Enteroviruses initiate entry into a host cell by binding to cell surface receptors and undergoing receptor-mediated endocytosis. Entry receptors vary between enteroviruses and include scavenger receptor B2 (SCARB2) and P-selectin glycoprotein ligand 1 (PSGL-1) for EV71 [46,47,48], the coxsackievirus and adenovirus receptor (CAR) for CVB [49,50,51], the poliovirus receptor (PVR/CD155) for PV [52], and the neonatal Fc receptor (FcRn) for echoviruses [53,54], amongst others (Table 1). In some cases, enteroviruses bind to additional attachment factors, the most common of which is decay accelerating factor (DAF)/CD55 [55,56]. Despite differences in cellular receptors, enteroviruses have generally well-conserved life cycles (Figure 2). In intestinal epithelial cell lines, CVB binding to DAF has been proposed to facilitate the induction of cell signaling from the apical domain, which in turn facilitates delivery of viral particles to their primary receptors [57]. Similarly, echovirus binding to DAF has also been proposed to facilitate entry into intestinal epithelial cell lines, although the role of intracellular cell signaling and the primary echovirus receptor in this process remains unclear [58].

After binding and entry, enteroviruses undergo uncoating in order to release the viral genome. Uncoating occurs either after the virus binds to the cell receptor or is initiated through a pH change in the endosome. This uncoating process allows the RNA genome to be released from the protective capsid into the cytoplasm or endosome. Several studies have investigated the speed at which PV virions uncoat in nonpolarized cells using either fluorescently-labeled capsids and viral RNA (vRNA) or neutral red-incorporated vRNA. These studies reveled that vRNA is released from the capsid within 30 minutes of entry [64,65]. However, other studies using polarized cells of the blood–brain barrier with apical and basolateral domains suggest that uncoating is a slower process that requires actin cytoskeleton remodeling [66]. Additionally, the speed of uncoating may differ between enteroviruses based on the requirements of attachment factors, such as DAF, or other cellular proteins required for entry [57].

Once viral RNA has entered the cytoplasm it is translated by host ribosomes (Figure 2). Historically, it was thought that the viral RNA was translated into a single polyprotein. However, a recent study discovered a second open reading frame (ORF) in some enterovirus genomes [67]. This study found a small ORF that is located in the 5’ end of the untranslated region and is suggested to be important in replication in intestinal epithelial cells [67]. Regardless of whether it is a single polyprotein or two proteins, the resulting product is then proteolytically cleaved by the viral proteases 2A and 3C. The resulting proteins include 10 proteins such as capsid proteins and other replication proteins including the RNA-dependent RNA polymerase 3D (3D^pol^). 3D^pol^ initiates the synthesis of the negative-stranded copy of the genome making a dsRNA intermediate, which becomes the template to generate new positive-stranded genomes. Replication of viral RNA occurs in replication organelles that derive from host membranes that are induced upon viral infection [68]. These replication organelles can protect the RNA and replication intermediates from cytosolic localized innate immune pattern recognition receptors (PRRs) that are important for the detection of foreign RNA [68]. The newly synthesized positive-stranded genome is packaged into virions for release out of the cell. The virions are assembled into protomers and pentamers using the capsid proteins VP0, VP1 and VP3. After the RNA is packaged into the virion, VP0 is processed into VP2 and VP4, which results in mature enterovirus virions [41,69].

Classically, enteroviruses have been thought to exit the cell through a lytic form of cell death where the cell undergoes lysis and releases the progeny virions to infect neighboring cells [41] (Figure 2). Recently, new studies have suggested that enteroviruses can also exit the host cell through non-lytic pathways [70,71,72]. These studies showed that during infection of PV and a related Picornavirus, hepatitis A virus, progeny virions are able to acquire host cell membranes to exit the cell in a vesicle in order to infect new cells [70,71]. These studies have shifted how enterovirus release is considered, but more work is needed to establish whether all enterovirus species are able to undergo non-lytic release and whether GI-derived cells permit this form of release.

## 3. Detection of Enteroviruses by Pattern Recognition Receptors

Detection of pathogens by the host immune system is an important first step in the clearance of viral pathogens. Previous studies have shown that echovirus 11 (E11), EV71, and CVB induce a robust innate immune response in intestinal epithelial cell lines and primary cells [73,74,75]. Viruses can be detected by the innate immune system in a variety of ways. PRRs are imperative in the detection and response to viral pathogens and are germ line encoded. PRRs detect pathogen associated molecular patterns (PAMPs) and respond by inducing an antiviral state. 

### 3.1. Detection by TLRs

One class of PRRs is Toll-like receptors (TLRs). TLRs are a class of 10 transmembrane PRRs that recognize a variety of PAMPs. Within the TLR family, two additional categories exist, which are TLRs that are localized to the cell surface as well as TLRs that are localized to the endosome [76]. Generally, TLRs have a PAMP binding domain on the N terminal region of the protein that is either on the extracellular domain or in the endosomal lumen and an intracellular signaling region on the C terminal end [77]. Here we will only discuss TLRs that detect RNA viruses but note other TLRs exist to sense DNA virus and bacterial derived PAMPs, such as TLR5 (flagellin), which have been extensively reviewed elsewhere [77,78,79].

TLR3 is primarily expressed on the endosome and recognizes dsRNA [80]. TLR3 is expressed under basal conditions in most cells and is not typically induced by interferon (IFN) or enterovirus infection [81]. TLR3 mediates an IFN response through Toll/IL-1 receptor domain-containing adaptor inducing interferon-beta (TRIF) and interferon regulatory factor (IRF)-3. The antiviral response mediated through TLR3 has been shown to be important in controlling PV, coxsackievirus A16 (CVA16), and coxsackievirus B3 (CVB3) [82,83,84]. In fact, the interferon stimulated genes (ISGs) that are produced by the induction of type I IFNs downstream of TLR3 are directly antiviral against CVA16. When expression of TLR3 is knocked out in mice, these animals have a more severe CVA16 infection compared to wild type control animals and develop severe paralysis and death [83]. Others have shown that TLR3 is also imperative for antiviral signaling during PV infection in mice [85]. Additionally, in vitro studies have shown that when cells are depleted of TRIF, a downstream adaptor molecule of TLR3, EV71 replication increases [86]. Although a rare polymorphism in TLR3 was identified in a patient who developed CVB-associated myocarditis, genetic variants in TLR3 or other IFN-associated factors are not commonly found in patients with viral-associated myocarditis [87,88]. Instead, these patients often express variants in genes associated with inherited cardiomyopathies, suggesting that TLR3 signaling is not the sole determinant of CVB-induced myocarditis. Nonetheless, in vitro and in vivo studies provide strong evidence that TLR3 is important for the detection and antiviral control of many enterovirus species.

Several studies provide support that TLR3 is an essential TLR in enterovirus infection. However, other TLRs can also play significant roles. Although TLR4 is thought to be key during bacterial infection since it mainly senses lipopolysaccharide (LPS), a protein found on gram negative bacteria, TLR4 plays an important role in secondary target tissues (tissues other than the route of entry) of enterovirus infection. TLR4 is localized to the cell surface where it can detect extracellular bacterial pathogens and has been shown to be important in myocarditis associated with CVB3 infection [89,90]. Studies have shown the TLR4 activation induces proinflammatory cytokines, which is seen in dilated cardiomyopathy, and a positive correlation between TLR4 and enterovirus RNA in endomyocardial biopsy tissues [90]. Furthermore, coxsackievirus B4 (CVB4) has been shown to induce proinflammatory cytokines through TLR4 in the pancreas, which leads to the progression of type I diabetes [91]. It is still unclear how TLR4 detects enterovirus infection; however, evidence points to a role of TLR4 in the induction of proinflammatory cytokines and clinical pathology during infection.

Both TLR7 and TLR8 sense ssRNA and are localized to the endosome [92]. Typically, these TLRs are not thought to be ISGs and; therefore, their expression is independent of IFN induction. However, a number of studies have demonstrated that TLR7 and TLR8 can be induced upon enteroviral infection. CVB3 can induce TLR7 and TLR8 expression after 48 h of infection at a low multiplicity of infection (MOI) [81]. Additionally, EV71 induces expression of TLR8 in cell lines and expression of TLR7 and TLR8 are increased in lung and brain tissues from children who died from EV71 infection [73,93]. Although the role of TLR7 and TLR8 have not been extensively studied during enterovirus infection, it is becoming clear that these PRRs may play key roles in the induction of proinflammatory cytokines. In fact, CVB is known to cause myocarditis due to chronic inflammation of the myocardium. This release of inflammatory cytokines has been linked to TLR8 and TLR4 [94,95]. These studies suggest that TLR7 and TLR8 play a significant role in enterovirus infection.

### 3.2. Detection by RLRs

TLRs play a key role in the detection of extracellular and endosomal localized pathogens, but RIG-I like receptors (RLRs) are arguably the crucial sensors for the detection of enteroviruses due to their localization to the cytoplasm. RLRs that are able to detect RNA virus infection are Retinoic acid-inducible gene I (RIG-I) and Melanoma differentiation-associated antigen 5 (MDA5). Both MDA5 and RIG-I have two caspase recruitment domains (CARD-like domains) at the N terminus as well as a DExD box RNA helicase, which is important for the detection of viral PAMPs [96]. RIG-I is a cytosolic PRR that recognizes RNA ligands such as 5’ triphosphate RNA (5’ pppRNA) [97]. In vitro data suggests that RIG-I is not always activated by enterovirus infection due to the VPg protein binding to the free 5’ triphosphate RNA, which would normally activate RIG-I [41]. However, recent studies have suggested a role of RIG-I in CVB3 infection [98,99]. Feng et al, suggests that the 5’ clover leaf of CVB3 is able to activate RIG-I since it contains triphosphate containing RNA [99]. However, this may be specific to CVB3 in cell line models since mice that are deficient in RIG-I have no difference in susceptibility to enterovirus infection compared to WT controls [82].

MDA5 detects long cytoplasmic dsRNA [100,101,102]. Several studies have indicated that MDA5 specifically interacts with enterovirus dsRNA, a replication intermediate, during CVA, CVB, EV71, and other enteroviruses [103,104,105,106]. Moreover, a polymorphism in MDA5 has been suggested to be a risk factor for more severe EV71 infection [107]. Children with this polymorphism exhibited more severe symptoms during EV71 infection compared to children without the polymorphism, suggesting a role of MDA5 in the detection of enterovirus infection. Furthermore, mice that are deficient in MDA5 are more susceptible to enterovirus infection and succumb to disease much more rapidly [105,108]. In addition to this increase in susceptibility, MDA5 deficient animals infected with CVB3 display severe hepatic necrosis of the liver [105]. Collectively, these studies point to the essential role of MDA5 in the detection of enteroviruses.

The adaptor protein for both RIG-I and MDA5 is the mitochondrial antiviral-signaling protein (MAVS), which is localized to the mitochondria and peroxisomes. When RIG-I or MDA5 are activated by dsRNA, the CARD domains become ubiquitinated [109,110], leading to the formation of MAVS aggregates in the mitochondrial membrane [111]. Aggregation of MAVS leads to the activation of NF-κB and IRF3, which then induce IFN [112]. In vitro studies have concluded that overexpression of MAVS can inhibit CVB3 replication by increasing the amount of IFN induction [113]. Although some studies have showed that MAVS deficient mice do not have an increased CVB3 viral load compared to WT controls, these animals succumb to infection much earlier than WT animals, suggesting that MAVS signaling and MDA5 dependent activation of IFN is imperative to host response to infection [105]. Overall, these PRRs and adaptor molecules have been shown to be imperative for sensing enterovirus infections.

## 4. Evasion of Innate Immunity by Enteroviruses

Viruses have evolved mechanisms to evade the induction of the antiviral state of the cell. Viral protease-mediated cleavage of PRRs allow enteroviruses to impact downstream signaling cascades, resulting in loss or reduced induction of IFN or interferon stimulated genes (ISGs). Table 2 summarizes these specific events and will be detailed below. As a result of these cleavage events, viruses are able to replicate more efficiently in the cell. Each species of enterovirus has developed its own set of mechanisms of evasion. Here, we will discuss current knowledge of evasion mechanisms of enteroviruses and how they antagonize host innate immune signaling.

### 4.1. Evasion of TLRs by Enteroviruses

**Figure 3:**
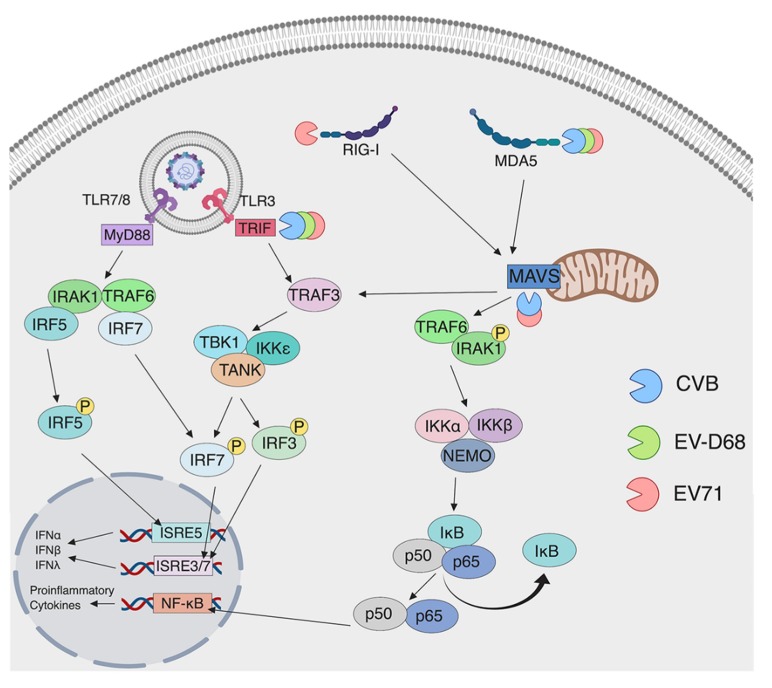
Enterovirus evasion strategies of PRR-mediated signaling. Enteroviruses target innate immune signaling proteins through cleavage by the viral proteases 2A and 3C. Shown are the targets for the CVB (blue), EV-D68 (green) and EV71 (red) proteases. All three viral proteases target MDA5 and the TLR adaptor protein, TRIF, as mechanisms to halt antiviral innate immune signaling in infected cells.

Enteroviruses are very efficient at disrupting downstream innate immune signaling. We have previously shown that, in human embryonic kidney cells (HEK293), CVB3 3C protease (3C^pro^) cleaves TRIF, a downstream adaptor molecule of TLR3 [114]. Cleavage of TRIF results in loss of TLR3-dependent induction of IFN and NF-κB. Other groups have shown that EV-D68 and EV71 3C^pro^ can also cleave TRIF resulting in decreased signaling downstream of TLR3 [115,116]. These studies, which include many different enterovirus species, show that TLR3-dependent IFN induction is antiviral against enteroviruses and is a key evasion target for these viruses (Figure 3). In addition to TLR3, TLR7 has been shown to be targeted by some enterovirus species, but the mechanisms that they use to target it are not well understood. As discussed previously, some enteroviruses, such as CVB3, can induce expression of TLR7 during infection. However, other enteroviruses seem to target TLR7. The detection of vRNA by TLR7 has been shown to increase autophagic flux [124]. In fact, one study showed that, in human bronchial epithelial cells (16HBE), TLR7-dependent type I IFN induction is reduced by EV71 and CVA16 [125]. This study concluded that autophagy induced by these viruses reduces endosome formation, resulting in the decreased expression of TLR7 to evade TLR7-dependent induction of autophagic flux in this cell type. This finding potentially demonstrates that some enterovirus species evade detection by TLR7, but others benefit from the induction of proinflammatory cytokines by TLR7.

### 4.2. Evasion of RLRs by Enteroviruses

In addition to TLRs, enteroviruses also target members of the RLR family for cleavage to evade innate immune signaling. MDA5, which is important for the sensing of enteroviruses in the host cell, is a target of viral proteases in many different studies. CVB3 2A^pro^ has been shown to cleave MDA5 in HeLa cells [121]. However, this study does not determine whether the cleavage products are still able to induce IFN signaling or whether cleavage of MDA5 hinders MDA5-depedent IFN induction. Similar studies using CVA16, CVA6, and EV-D68 have indicated that 3C^pro^ cleaves MDA5 [120]. Although the authors show that IFN signaling is disrupted when cells were transfected with 3C^pro^, they do not specifically show that the cleavage products are not functional in inducing an IFN response. Additionally, MDA5 has been shown to be cleaved in PV-infected HeLa cells [122]. However, unlike the prior studies, this study concluded that the cleavage was not dependent on viral proteases but was instead mediated by cellular caspases activated during infection [122]. Furthermore, EV71 is able to cleave MDA5, but the mechanism is less clear [104]. Apart from the different mechanisms enteroviruses use to disrupt MDA5 signaling, infected cell lines have been shown to have cleavage products resulting in the inhibition of IFN induction.

RIG-I has also been shown to be cleaved in cells infected with different enteroviruses. Since RIG-I mainly detects 5’ pppRNA, the reason why enteroviruses would target this RLR is not well understood but, as discussed before, new evidence suggests that RIG-I may detect enteroviruses (see Section 3.2). PV 3C^pro^ is able to cleave RIG-I in infected HeLa cells by 6 h post infection [119]. In addition to viral protease-mediated cleavage of these sensors, EV71 alters IFN induction by targeting the ubiquitination of RIG-I [117], which is critical for downstream signaling [117,118]. Previous studies have shown that CYLD (cylindromatosis), a deubiquitinating enzyme, is a negative regulator of RIG-I [126]. During viral infection, a cellular microRNA, miR-526a, is upregulated and induces the downregulation of negative regulator, CYLD, leading to enhanced signaling of RIG-I. However, EV71 is able to downregulate miR-526a resulting in normal levels of CYLD [127]. As a result, RIG-I ubiquitination decreases inhibiting IFN induction. Further research to delineate the specific enterovirus PAMP that RIG-I is able to detect to induce IFN and the mechanisms enteroviruses use to target RIG-I will be needed to understand this aspect of enterovirus infection.

Numerous studies have investigated the viral protease mediated cleavage of MAVS. Targeting MAVS, the adaptor protein of RIG-I and MDA5, ablates IFN induction of both RIG-I and MDA5, making this protein an essential target of many enteroviruses. CVB3 2A^pro^ and 3C^pro^ cleave MAVS in various cell lines [114,121]. The resulting cleavage products are nonfunctional and are deficient in NF-κB and IFN signaling [114]. EV71 2A^pro^ is able to cleave MAVS in HeLa cells [121,123]. These studies showed that, similar to CVB infection, the products of MAVS in EV71-infected cells are deficient in NF-κB and IFN signaling [121,123]. However, since these studies are mainly performed in cell lines, further research is needed to determine if enteroviruses behave similarly in primary cells such as those of the GI tract.

## 5. Models to Study Enteroviruses in the Gut

### 5.1. In Vitro and Ex Vivo Models to Study Enterovirus Infection

Many different models to study enteroviruses in the GI tract exist. These include cell lines, three-dimensional cell culture-based models, mouse models, and non-human primate models. Cell lines that model the GI tract and have been applied to enterovirus research include Caco-2, HT-29, T84, MODE-K (murine), and IEC-6 (rat) cells. The main cell line that has historically been used to model enteroviral infections of the human intestinal epithelium is Caco-2 cells. Caco-2 cells have characteristics of enterocytes, which includes a brush border and tight junctions [128]. In addition to standard culture systems that utilize Caco-2 cells, we have also developed a three-dimensional culture model using Caco-2 cells grown on beads in a rotating wall vessel bioreactor that exhibit the properties of the intestinal epithelium, and have applied this system to model enterovirus infections in the GI tract [129].

Other 3D culture model systems include organoids. Organoids are 3D enterospheres that are derived from pluripotent stem cells or embryonic stem cells [130]. Organoids are spherical structures that are hollow in the middle, have apical and basolateral polarity, and form a spherical layer of epithelium [131,132]. Pluripotent stem cells are differentiated into ectoderm, then hindgut ectoderm, and finally form spheroids with the addition of correct growth factors [130,133]. In addition, organoids contain a mesenchymal cell layer that develops under the organoid. Studies have shown that organoids are able to differentiate into the absorptive and secretory lineages of the GI epithelium [130]. However, this culture model has not yet been applied to enterovirus research.

**Figure 4:**
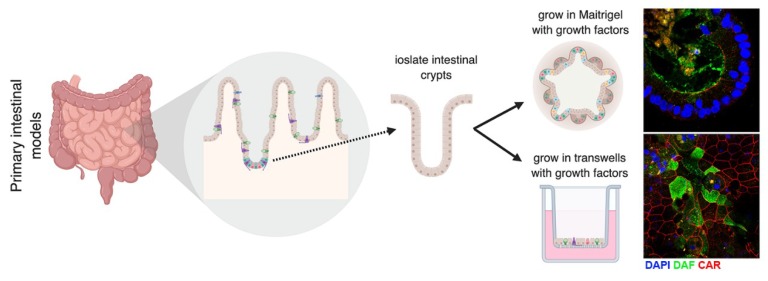
Primary intestinal models to study enterovirus infection. Intestinal crypts are isolated from the small intestine and plated either in Matrigel or on transwells. Crypts plated in Matrigel form a 3D structure called enteroids. When plated in Matrigel, enteroids have an “inside-out” structure, where the apical domain faces inward, and the basolateral domain is facing outward. When crypts are plated on transwells, they form a monolayer that has apical and basolateral polarity. Images shown are from crypts that were plated in Matrigel (top) or on a transwell (bottom) and stained with DAF (green) and CAR (red), which are involved in CVB attachment, uncoating, and entry.

In addition to organoids, enteroids are used as another 3D system to model the GI epithelium. Enteroids are formed through the isolation of intestinal crypts from whole human and murine intestinal tissues [134], which contain LGR5+ stem cells [33]. The crypts can be isolated and plated in Matrigel, where they form 3D spherical enteroids, or on transwells, where they form 2D monolayers that exhibit barrier function (Figure 4) [74,75,135]. Enteroids that are plated in matrigel have an “inside-out” phenotype, where the apical surface is facing into the lumen and the basolateral surface is on the outside of the structure [75]. This makes the Matrigel model a difficult model for studying viruses that use receptors that are localized to the apical surface, since the apical surface is not accessible without disruption of the 3D nature of these structures. To overcome this limitation, we and others have developed a transwell-based model system in order to gain access to both the apical and basolateral surfaces [75,136,137]. These transwell models allow for infection at either the apical or basolateral surfaces and for collection of growth medium from these distinct compartments. We have applied both Matrigel- and transwell-based enteroid models to study enterovirus–GI interactions (Figure 4). Using these systems, we have identified differences in the cell-type specificity by which enterovirus target the GI epithelium. For example, whereas E11 preferentially infects enterocytes and enteroendocrine cells, EV71 replication is largely restricted to goblet cells [74,75]. In addition, using a transwell-based model, we have shown that enteroviruses also exhibit differences in the polarity by which they enter into and egress from the epithelium, with E11 exhibiting a basolateral polarity of entry and a bidirectional manner of egress whereas EV71 both enter and is released preferentially from the apical domain [75]. Perhaps most striking in these models is the robust antiviral response elicited in response to enterovirus infections. In contrast to most cell lines, which induce little to no IFN signaling, primary human enteroid models potently induce an antiviral response to enteroviral infections [75]. Perhaps not surprisingly given their role in barrier defenses, these models almost exclusively induce antiviral type III IFNs in response to infection. Collectively, these data highlight the potential relevance of primary-based intestinal cell systems to model enterovirus infections. Although these in vitro models recapitulate the multicellular complexity of the small intestine, making them a more physiologically relevant model compared to cell lines, they do; however, lack bacterial interactions, which can impact enteroviral infection. In vitro studies have shown that PV virions can bind to bacteria and that some bacterial strains can facilitate enterovirus infection [138]. In fact, bacteria can aid in co-infection of different enteroviruses, which allows for genetic recombination [138]. Other studies have shown that certain species of bacteria can increase thermal stabilization of PV and CVB [139]. This leads to the question of whether other enteroviruses are also impacted by bacterial co-infection and what impact this has on pathogenesis in vivo. Studies investigating the role of the microbiome on enteroviruses are imperative to understand in vivo pathogenesis.

### 5.2. In Vivo Models to Study Enterovirus Infection

While the above-described in vitro models have provided many insights into various aspects of enterovirus infections of the GI tract, in vivo models are also needed to understand complex interactions that occur during enteroviral infections, such as the interaction of viral particles with bacteria or the complex interaction with the immune system. One of the first mouse models to study enterovirus infection was the transgenic PV receptor mouse. The authors demonstrated that mice expressing the human homologue of the poliovirus receptor (PVR) were able to be infected with PV through intracerebral injection, where they displayed signs of paralysis similar to human disease [140]. Due to the route of infection, animals did not need to be immunosuppressed such as blocking and depleting type I interferon receptor (IFNAR). Since this model was established, others have developed models for other enterovirus infection using a variety of different methods. Many mouse models of enterovirus infection use “humanized” mice that express the human form of the viral receptor. Since mice are not the natural host of enteroviruses, the mouse homologs of the entry receptors are often not sufficient for infection or the affinity of viruses much less. Several groups have used this strategy, including generating “knock-in” animals expressing human SCARB2 for EV71 infection [141]. Very commonly, ablation of IFNAR or oral infection at high viral doses is required to generate in vivo mouse models of enteroviruses [142,143,144]. These strategies allow the infection of enteroviruses in mice, which normally do not support robust replication. However, many of these models are based on IP injection or other non-oral infection routes. However, this route of administration bypasses the primary site of infection observed in humans. Thus, models that include oral infection are imperative to understand how enteroviruses infect the GI tract and disseminate into secondary target tissues causing clinical disease.

Several models of oral infection have been established for a E11, PV, CVB, and EV71, and have been shown to recapitulate human disease [53,143,145,146,147]. An adult model of oral infection of CVB using IFNAR-deficient mice investigated the pathogenesis of a mutant CVB virus that emerged after passage through a mouse that exhibited a large plaque phenotype [142]. In addition to adult mouse models of oral infection, several studies have established neonatal infection models for a number of enteroviruses. We recently established a neonatal model for E11 infection by the enteral route in human transgenic mice expressing the human homolog of FcRn [53] and showed that only transgenic mice exhibited viral replication in the small intestine, liver, and blood seven days post oral infection [53]. An oral infection model of PV was established using transgenic animals expressing the human homolog of PVR and using IFNAR-deficient mice [143]. Neonatal, transgenic, IFNAR-deficient mice infected with PV exhibited viral replication in the blood and small intestine two and three days post oral infection [143]. In addition to E11 and PV, multiple neonatal models of oral EV71 infection have been established [146,147]. One study established an oral infection model using chimeric receptor-expressing transgenic mice, showing that oral infection of clinical isolates of EV71 leads to viral replication in the stomach, small intestine, colon, and brain seven days post infection [146]. Another model of EV71 infection using outbred mice showed seven-day-old outbred mice that were orally infected with EV71 displayed skin rashes early during infection, which progressed to hind limb paralysis [147].

In addition to mouse models, several studies have used non-human primate models to study EV71, CVB, and PV pathogenesis. One study showed that rhesus monkeys can be infected with EV71 through the intravenous, respiratory, and oral routes but had limited viral replication in the blood after intracerebral infection [148]. This study showed that EV71 disseminated to the brain and causes neuropathological damage [148]. Moreover, oral infection models of EV71 have been established in cynomolgus monkeys that showed degeneration and necrosis of neurons in the central nervous system of infected monkeys [149]. Additionally, neonatal rhesus monkeys animals infected with EV71 show signs of clinical hand-foot-and-mouth disease as seen in humans [150]. Furthermore, a model of CVB-induced myocarditis was established using cynomolgus monkeys which exhibited viral myocarditis similar to human disease. This study showed that, following intravenous inoculation with CVB, animals experienced myocardial injury and inflammatory cells infiltration in the heart of infected animals [151]. Another study using patas monkeys showed that intravenous CVB infection caused abnormalities in blood glucose as well as impaired insulin secretion [152]. In addition to EV71 and CVB, several models to study PV have been established which include oral, subcutaneous, intravenous, intraspinal, and intracerebral infection [153,154,155,156,157]. These models were incredibly important for understanding the immune response to the PV vaccine [17,158]. One study showed that rhesus, cynomolgus, and bonnet macaques were all susceptible to oral PV infection [156]. When these macaques were fed PV, they developed paralysis [156]. Other studies have shown that infant cynomolgus monkeys that were fed PV developed paralytic poliomyelitis [159]. Together, the use of in vitro models and in vivo models, including mouse models and non-human primate models, will aid in our understanding of enterovirus entry, detection by the host immune response, and evasion mechanisms these viruses use to subvert the innate immune response.

## 6. Concluding Remarks

Enteroviruses remain a significant global public health concern. The field has made significant progress in determining how enteroviruses are detected by host cells and the mechanisms they use to evade this detection. With the continued development of in vitro, ex vivo, and in vivo models that fully recapitulate the GI epithelium, we will gain a better understanding of the mechanisms used by enteroviruses to breach the intestinal barrier. These models could also facilitate the development of novel therapeutic targets and/or strategies to prevent or treat enterovirus infections and ultimately alleviate morbidity and mortality caused by these infections.

## Figures and Tables

**Table 1:** Enterovirus receptors and attachment factors.

Host Protein	EV Serotype	Role	Reference
PVR (CD155)	PV	Binding, entry, uncoating	[52]
CAR	CVB1, CVB2, CVB3, CVB4, CVB5, and CVB6	Binding, entry, uncoating	[49,50,51]
DAF	CVA21, CVB1, CVB3 (some isolates), CVB5, E3, E6, E7, E11, E12, E13, E19, E19, E20, E21, E25, E29, and E30	Attachment	[55,56]
SCARB2	EV71, CVA7, CVA14, and CVA16	Binding, entry, uncoating	[46]
PSGL1	EV71, CVA2, CVA7, CVA10, CVA14, and CVA16	Attachment	[47,59]
KREMEN1	CVA10	Binding, entry	[60]
Sialic acid	EV71	Attachment	[61]
ICAM5	EV-D68	Binding, entry	[62]
Integrin ⍺_2_β_1_ (VLA-2)	E1	Binding, entry, uncoating	[63]
FcRn	Echoviruses	Binding, entry, uncoating	[53,54]

**Table 2:** Enterovirus targets of PRRs.

Host Protein	EV Serotype	Mechanism of Cleavage	Reference
TRIF	CVB3	3C^pro^	[114]
	EV-D68	3C^pro^	[115]
	EV71	3C^pro^	[116]
RIG-I	EV71	Decreases ubiquitination of RIG-I inhibiting recruitment to MAVS	[117,118]
	PV	3C^pro^	[119]
MDA5	CVA6	3C^pro^	[120]
	CVA16	3C^pro^	[120]
	CVB3	2A^pro^	[121]
	EV-D68	3C^pro^	[120]
	EV71	Unknown	[104]
	PV	Caspase Dependent	[122]
MAVS	CVB3	2A^pro,^ 3C^pro^	[114,121]
	EV71	2A^pro^	[121,123]
